# Education policies to increase rural physicians in Japan: a nationwide cohort study

**DOI:** 10.1186/s12960-021-00644-6

**Published:** 2021-08-24

**Authors:** Masatoshi Matsumoto, Yasushi Matsuyama, Saori Kashima, Soichi Koike, Yuji Okazaki, Kazuhiko Kotani, Tetsuhiro Owaki, Shizukiyo Ishikawa, Seitaro Iguchi, Hitoaki Okazaki, Takahiro Maeda

**Affiliations:** 1 Department of Community-Based Medical System, Graduate School of Biomedical and Health Sciences, Hiroshima University, 1-2-3 Kasumi, Minami-ku, Hiroshima, 734-8551 Japan; 2 Medical Education Center, Jichi Medical University, 3311-1 Yakushiji, Shimotsuke, 329-0498 Japan; 3 Environmental Health Sciences Laboratory, Graduate School of Advanced Science and Engineering, Hiroshima University, 1-5-1 Kagamiyama, Higashi-hiroshima, 739-8529 Japan; 4 Division of Health Policy and Management, Center for Community Medicine, Jichi Medical University, 3311-1 Yakushiji, Shimotsuke, 329-0498 Japan; 5 Kitahiroshimacho Yahata Clinic Nishiyawatahara 1453, Kitahiroshima-cho, Yamagata-gun, Hiroshima, 731-2552 Japan; 6 Division of Community and Family Medicine, Center for Community Medicine, Jichi Medical University, 3311-1 Yakushiji, Shimotsuke, 329-0498 Japan; 7 Education Center for Doctors in Remote Islands and Rural Areas, Graduate School of Medical Sciences, Kagoshima University, 8-35-1 Sakuragaoka, Kagoshima, 890-8544 Japan; 8 Department of Community Medicine, Niigata University Graduate School of Medical & Dental Sciences, 757 Asahimachidori-ichibancho, Chuo-ku, Niigata, 951-8510 Japan; 9 Department of General Medicine, Nagasaki University Graduate School of Biomedical, Sciences 1-7-1 Sakamoto, Nagasaki, 852-8501 Japan

**Keywords:** Rural health, Health workforce, Health policy, Education, Japan

## Abstract

**Background:**

Japan has established comprehensive education-scholarship programs to supply physicians in rural areas. Their entrants now comprise 16% of all medical students, and graduates must work in rural areas for a designated number of years. These programs are now being adopted outside Japan, but their medium-term outcomes and inter-program differences are unknown.

**Methods:**

A nationwide prospective cohort study of newly licensed physicians 2014–2018 (*n* = 2454) of the four major types of the programs—Jichi Medical University (Jichi); regional quota with scholarship; non-quota with scholarship (scholarship alone); and quota without scholarship (quota alone)—and all Japanese physicians in the same postgraduate year (*n* = 40,293) was conducted with follow-up workplace information from the Physician Census 2018, Ministry of Health, Labour and Welfare. In addition, annual cross-sectional survey for prefectural governments and medical schools 2014–2019 was conducted to obtain information on the results of National Physician License Examination and retention status for contractual workforce.

**Results:**

Passing rate of the National Physician License Examination was highest in Jichi, followed in descending order by quota with scholarship, the other two programs, and all medical graduates. The retention rate for contractual rural service of Jichi graduates 5 years after graduation (*n* = 683; 98%) was higher than that of quota with scholarship (2868; 90%; *P* < 0.001) and scholarship alone (2220; 81% < 0.001). Relative risks of working in municipalities with the least population density quintile in Jichi, quota with scholarship, scholarship alone, and quota alone in postgraduate year 5 were 4.0 (95% CI 3.7–4.4; *P* < 0.001), 3.1 (2.6–3.7; < 0.001), 2.5 (2.1–3.0; < 0.001), and 2.5 (1.9–3.3; < 0.001) as compared with all Japanese physicians. There was no significant difference between each program and all physicians in the proportion of those who specialized in internal medicine or general practice in postgraduate years 3 to 5

**Conclusions:**

Japan’s education policies to produce rural physicians are effective but the degree of effectiveness varies among the programs. Policymakers and medical educators should plan their future rural workforce policies with reference to the effectiveness and variations of these programs.

**Supplementary Information:**

The online version contains supplementary material available at 10.1186/s12960-021-00644-6.

## Background

Countries around the world are concerned with the geographic imbalance in the supply of physicians [1], and policies to address it is required for all Member States of the World Health Organization to achieve universal health coverage [2]. For its part, the Japanese government has spent years formulating policies to increase and secure the number of physicians in rural areas. These policies had two aims: to increase the number of physicians nationwide, and to produce physicians who practice in rural areas. A typical example of the former is the "one prefecture one medical school" policy of the 1970s and 1980s, which has nearly doubled the total number of medical schools, and rapidly increased the number of physicians. Representative of the latter is Jichi Medical University (Jichi), a special medical school founded in 1972 to produce rural physicians [3–5].

Each policy was effective on its own, but not effective enough to redress the urban–rural imbalance of physician supply across Japan [6, 7]. Instead, the distribution of physicians had worsened by the time of the reform of postgraduate clinical training program, which became nationwide in 2004 [8–11]. Under pressure from rural prefectures, some medical schools, in cooperation with the prefectural and national governments, established *chiikiwaku*, or the regional quota program in 2008. The regional quota has rapidly spread to most of the medical schools in Japan [12–14]. The regional quota and Jichi have been reported as a Japanese original policy for recruiting and retaining a rural health workforce [3, 15].

The 47 prefectures established Jichi in partnership with the national government. It has produced more than 4,000 physicians over the past half century [16]. It takes two or three applicants from each of Japan’s 47 prefectures. After 6 years of a rural-oriented undergraduate education and passing the National Physician License Examination, all the Jichi graduates are required to work in their home prefectures for 9 years, which include 2 years of postgraduate training and about 5 years of rural service. By completing the 9 years of service, the undergraduate tuition of the graduates is forgiven. The number of Jichi graduates working in rural areas was reportedly 13 times higher under the obligatory service and four times higher after completing the service than non-Jichi graduates [4]. Jichi is now used as a reference case for rural health workforce policies in the world [17].

Based on the Jichi's success, most Japanese medical schools started to accept some of their entrants into a Jichi-like regional quota program. The regional quota is one of the largest policies that Japan has ever adopted to redress the geographic maldistribution of physicians [15]. Now 16.2% of medical students nationwide are quota entrants [14]. Typical quota programs have the following characteristics: applicants are limited to those who are legal residents of the prefecture in which the medical school is located; they undergo a special admission process; their undergraduate medical education has an emphasis on rural practice; and upon graduation they agree to work in the prefecture for a specified length of time. Most of the quota programs are bundled with a prefecture scholarship for their 6-year undergraduate education. In exchange, they are obliged to work in the prefecture for 9 years. More than half (54%) of them must work for 3–4 years in rural municipalities in the prefecture [18]. However, 20% of the regional quotas are not bundled with the scholarship. Graduates of these "quota alone" programs do not have the obligation but have an agreement, on admission, to practice in rural areas after graduation. Apart from scholarships bundled with the regional quotas, many prefectures have unbundled scholarship programs. These "scholarship alone" programs are available to students who entered medical schools through the usual admission process and hope to work somewhere in the prefecture. The scholarship amount is usually the same as the one offered to quota students with scholarships, as are the length and obligatory service requirements. A past study reporting short-term outcomes of quota and scholarship programs showed that the proportions of their graduates working in rural areas were higher than that of other physicians [14].

No study has directly compared the outcomes of Jichi, quota with scholarship, quota alone, and scholarship alone. Revealing each policy's advantages and disadvantages leads to the evidence-based policy making (EBPM) that the Japanese government is promoting [19]. Such a comparative study will also provide scientific evidence for policies of other countries, such as South Korea, which are introducing education-scholarship programs similar to Japanese regional quota with scholarship and Jichi [20–22]. Such evidence may be useful to some countries, including the United States, that have undergraduate medical education programs designed to produce rural physicians [23–26], and many countries, again including the United States, that offer financial incentive programs for medical students by a government or by other administrative bodies in exchange for practicing in rural areas [27, 28]. However, these education or financial incentive programs are different from the Japanese initiatives in that the latter contain both the undergraduate education and postgraduate placement. The undergraduate programs in other countries recruit students with rural background and provide rural-oriented medical education, but usually do not control the practice location of their graduates. The financial incentive programs do control the practice location, but do not engage with medical school admission process or undergraduate education. In contrast, the Japanese programs combine these two systems and provide continuous support from medical school admission to the end of postgraduate rural service. For both Jichi and the quota with scholarship, medical schools take the main responsibility in undergraduate education and prefectures are in charge of assigning the graduates; both medical schools and prefectures work closely together in managing the comprehensive education-scholarship programs. In this sense the education-scholarship programs such as Jichi and regional quota are a unique in means of producing rural physicians.

This is a nationwide cohort study that shows the most up-to-date and comprehensive outcomes of these comprehensive education-scholarship programs in Japan. We also compare the results among the four major programs, and through those comparisons, reveal each program's effectiveness. Then we discuss the advantages and disadvantages of each program in light of policy making and education planning.

## Methods

### Study design

This study is a combination of a prospective cohort study of quota and scholarship graduates, a retrospective cohort study of Jichi graduates, and a cross-sectional survey of prefectures and medical schools.

### Definition of quota and scholarship

This study defines a quota student as one whose "geographic background or location of graduated high school is restricted, and/or working place or specialty after graduation is specified." A prefecture scholarship is defined as “a scholarship which is given to a medical student and does not need to be repaid if the student works in an area designated by the prefecture for a certain term after graduation.

### Cohort of quota graduates and scholarship recipients

**Fig. 1:**
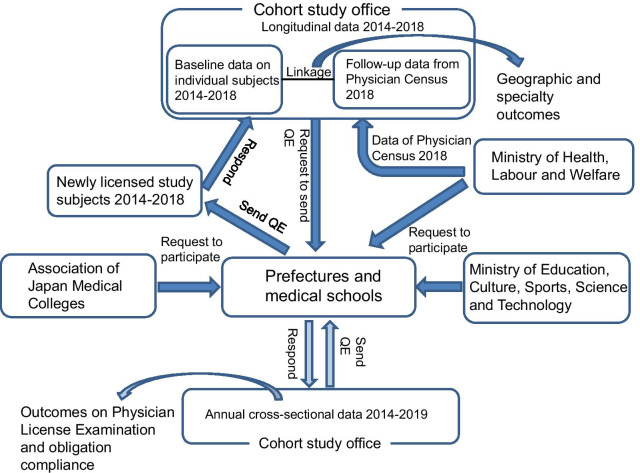
Design of cohort study for quota and scholarship subjects. *QE* questionnaire

The Japanese Council for Community-based Medical Education (JCCME) conducted a nationwide cohort study from 2014 to 2019. The groups of subjects were quota graduates with scholarship, non-quota graduates with scholarship (scholarship alone), and quota graduates without scholarship (quota alone). All subjects were physicians licensed in 2014–2019. All the graduates of medical schools in these years registered in the Survey of Physicians, Dentists and Pharmacists (Physician Census) 2018, Ministry of Health, Labour and Welfare constitute the comparison group. Details of the data collection process were reported previously [12–14], and are illustrated in Fig. 1 and noted in Additional file 1 in this paper.

### Cohort of Jichi Medical University graduates

Data of Jichi graduates was collected separately. Since 1972, Jichi has administered an annual survey to its graduates in all prefectures on their place of work, specialty, and status of obligation compliance. All the data were connected, on an individual basis, through the identification number of each graduate. The complete cohort data set is housed in the Institutional Research Office. For this study, information on the result of National Physician License Examination and obligation compliance status of those who graduated between 2014 and 2019, and the municipality code of the workplace and specialties in 2018 were extracted for analysis.

### Annual cross-sectional survey of prefectures and medical schools

Each June, the cohort office sends a questionnaire to each prefectural government and medical school to obtain information on the number of new graduates who had received scholarship (prefecture), who passed the National License Examination for Physicians (prefecture and medical school), and who have bought out the scholarship to free themselves from the contractual rural service (prefecture).

### Outcome measurement

We calculated the passing rate of the National Physician License Examination and retention rate for the contractual workforce in each group based on the results of the annual cross-sectional survey, and in-prefecture retention rate, the median population density of workplace municipality, distribution among population density quintiles, non-metropolitan rate, and internal medicine/general practice rate based on the results of the cohort data. All the outcomes of Jichi graduates were calculated directly from the Jichi cohort data.

The passing rate of the National Physician License Examination is the percentage of medical school graduates who passed the examination among all who took the examination. The retention rate for the contractual workforce (non-buying-out rates) is the percentage of graduates who did not buy out the received scholarship (or tuition, for Jichi graduates) and comply with the terms of being admitted to a quota and/or receiving scholarship. Geographic outcomes were measured as follows. Japan has three levels of government: municipal, prefectural, and national. We extracted data on municipal (city, town, village) populations and land areas in 2016 [29]. The physician data were connected to these municipality-based population data through the municipality code.

We first calculated the percentage of graduates who worked in the designated prefecture in which they were obliged to work (in-prefecture retention rate). Then, we calculated the proportion of those who worked in rural municipalities. Japan does not have an authorised and uniform definition of rural areas; therefore, we used two ways of gauging rurality for this study, both of which have been used in previous studies [14, 30, 31]. First all the municipalities (*n* = 1896) were divided into five quintile groups so that each quintile includes 20% of all the physicians of postgraduate year (PGY) 1 to 5. The cut-off values of population density for the five quintiles were 522.7, 1301.0, 4665.3, and 10,492.6 persons per square kilometre. Another way of showing rurality was to divide municipalities into metropolis and non-metropolis. A metropolis consists of all of the wards (*ku*) of the ordinance-designated cities (*seirei-shitei-toshi*), 23 special wards of Tokyo, and centre cities (*chukaku-shi*) (n = 257). All the other municipalities were treated as non-metropolis (n = 1639). This classification of municipalities is administratively used based on the Local Autonomy Act. We also calculated the proportion of those who specialized in internal medicine or general practice, which are Japan's two major primary care specialities.

### Statistical analysis

The retention rate of each graduation year cohort for contractual workforce was calculated with the direct method of survival analysis. The retention rate of all those who graduated between 2014 and 2019 was calculated with the Kaplan–Meier survival analysis in which subjects with various observation periods can be analysed. The rates were compared among Jichi, quota with scholarship and scholarship alone with the log-rank test. Mann–Whitney *U* test was used to compare median population densities. Comparison of the proportions of the categorical variables between groups was conducted with *χ*^2^ test or with Fisher's exact test. All the *P* values were adjusted with the Bonferroni correction for multi-comparisons in which more than three sub-groups were compared. Relative risks (RR), which were risk ratios, and their confidence intervals (CIs) were estimated using the unconditional maximum likelihood (Wald). Any *P* value less than 0.05 (two-sided test) was regarded as statistically significant. All statistical analyses were conducted with SPSS statistical software, version 24 (IBM-SPSS, Tokyo, Japan) and R version 4.0.2 software (R Foundation for Statistical Computing, Vienna, Austria).

## Results

Between 2014 and 2019, there was a cumulative total of 270 prefectures and 158 medical schools with eligible subjects. Among them, 268 prefectures (99.3%) and 158 medical schools (100%) responded to the annual cross-sectional survey, and sent questionnaires to potential subjects. The number of potential quota-alone subjects (2014–2018) was 1467; scholarship alone, 1751; and quota with scholarship, 2162. Of these, 508 (34.6%), 655 (37.4%), and 864 (40.0%) responded and were enrolled in the cohort, respectively. Responders whose physician identification numbers were missing or could not be connected to those in the 2018 Physician Census were excluded. Therefore, data for 483, 595, and 817 participants were subject to longitudinal analysis. All (100%) the Jichi graduates (559) were included in the Jichi cohort data set and analysed.

**Fig. 2:**
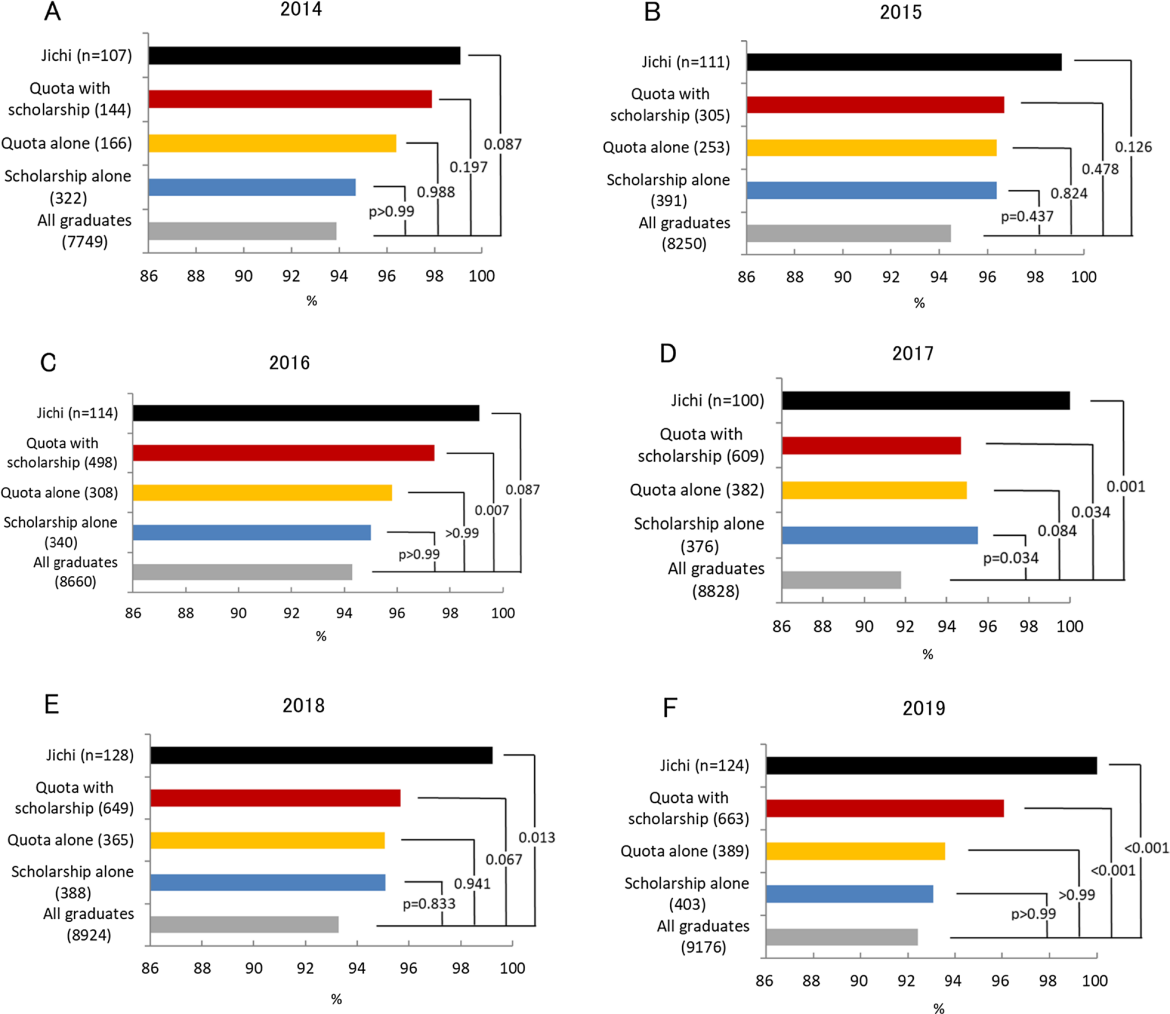
Passing rate of the National Physician License Examination. *Jichi* Jichi Medical University. *P* value was calculated with Fisher’s exact test with Bonferroni adjustment

Figure 2 compares the passing rates of the National Physician License Examination. A consistent pattern with Jichi in the first place and quota with scholarship in the second was observed throughout the years. The difference in rate between Jichi or quota with scholarship and all graduates was significant in 2016–2019.

**Fig. 3:**
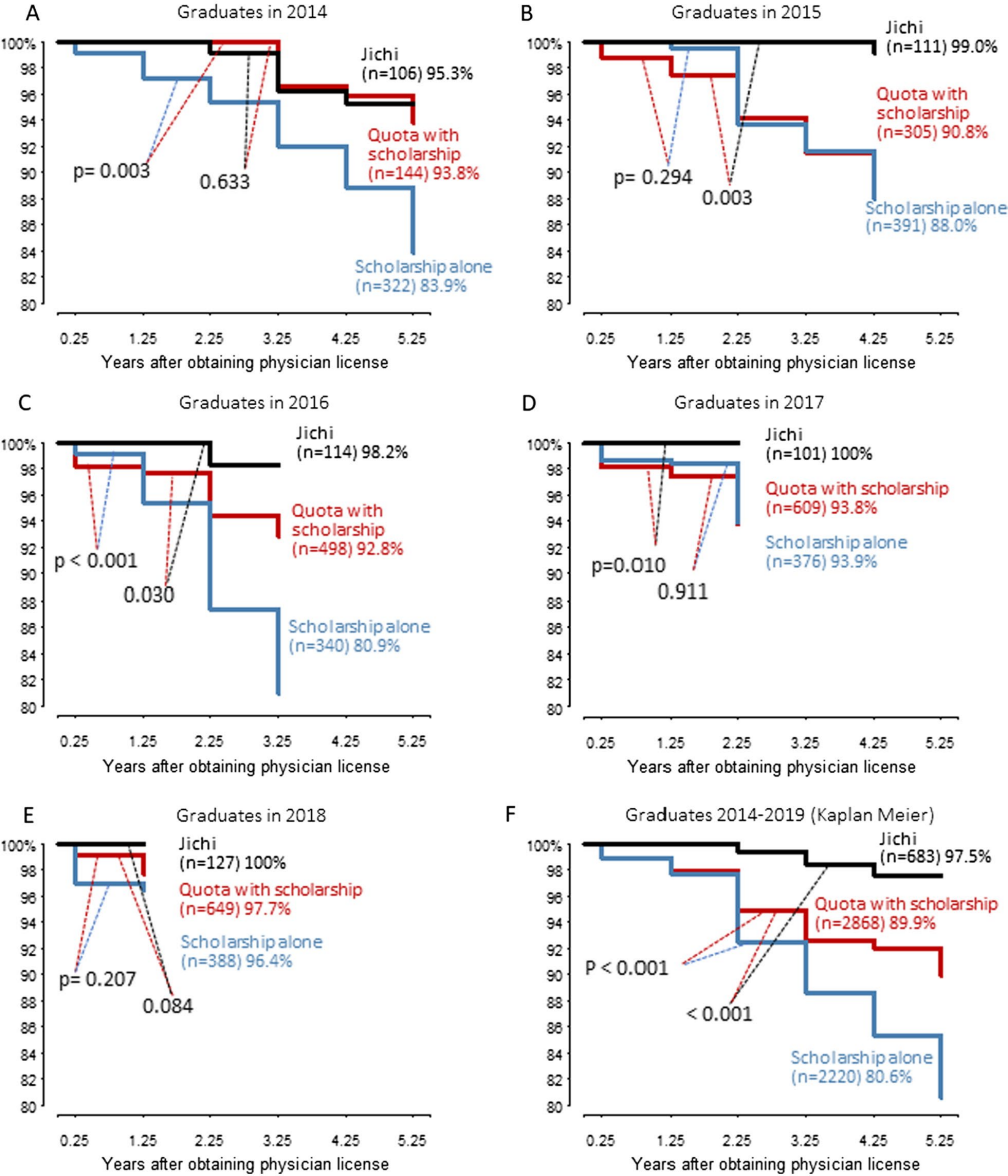
Retention rate for contractual workforce. The retention rate is the percentage of graduates who did not buy out the received scholarship (or for Jichi student, tuition). The retention was calculated in the direct method of survival analysis except for those who graduated between 2014 and 2019 (F) which was calculated with the Kaplan–Meier method

The retention rates for contractual workforce of each graduation year cohort (Fig. 3a–e) and of the cohort including all subjects (Fig. 3f) are shown. The contract retention rate of Jichi graduates 5 years after graduation (*n* = 683; 98%) was higher than that of quota with scholarship (2868; 90%; *P* < 0.001); the latter nevertheless was higher than that of scholarship alone (2220; 81% < 0.001).

**Fig. 4:**
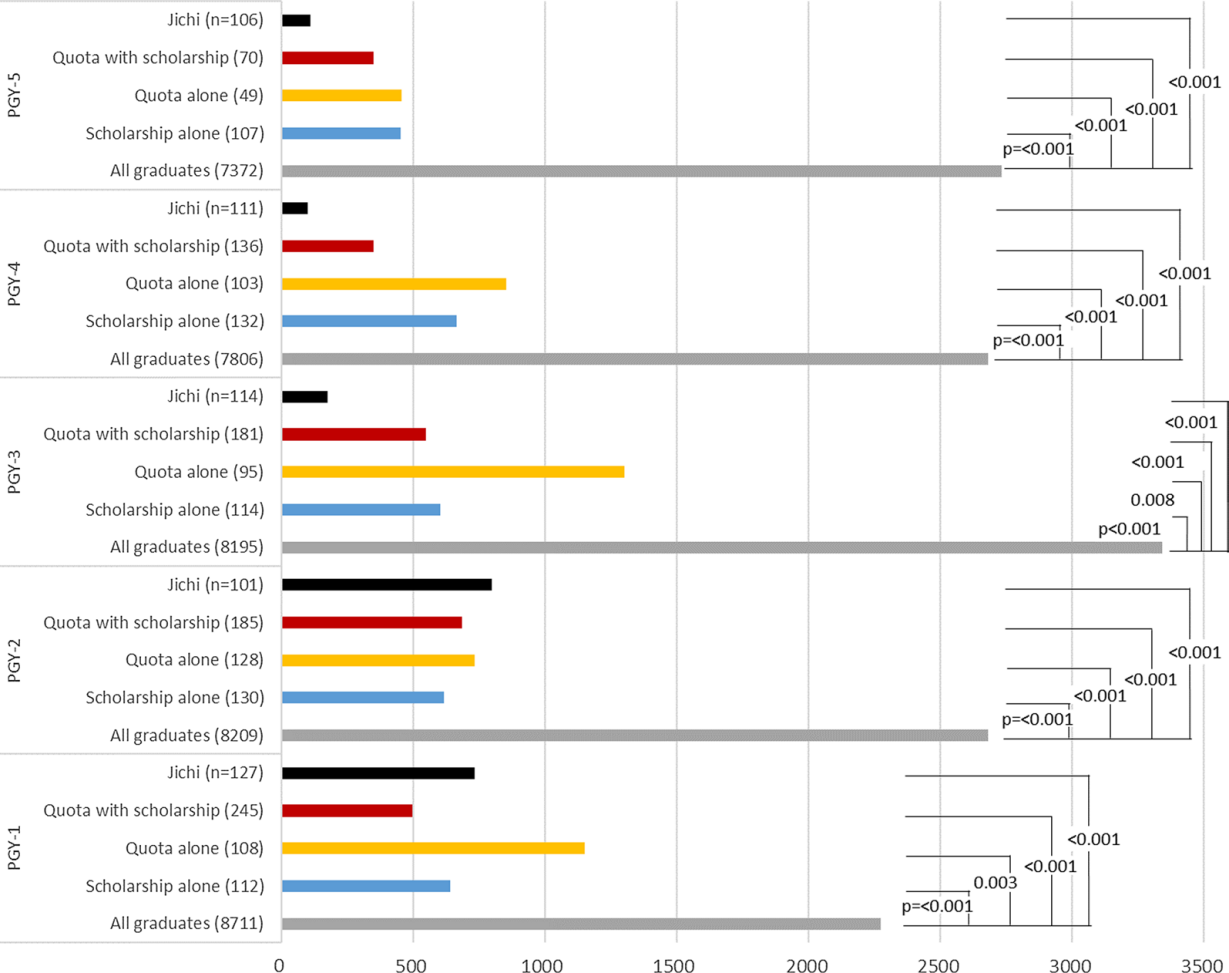
Median population density of workplace municipality. P value was calculated with Mann–Whitney *U* Test with Bonferroni adjustment

Comparisons of the median population density (Fig. 4) and the population density quintile (Table 1) of workplace municipality are shown. The median workplace population density of Jichi, quota with scholarship, quota alone and scholarship alone groups were significantly less than that of all graduates. The gap tended to be wider with the increase of PGY. The likelihood (relative risk) of working in the quintile of the least population density was highest in Jichi, followed by quota with scholarship, scholarship alone, and quota alone. In PGYs 1 and 2 (under postgraduate clinical training without rural service), the likelihoods are not substantially different among the four groups, but in PGYs 3 to 5 (potentially under rural service), the likelihood of Jichi stood out, followed by that of quota with scholarship and scholarship alone.

**Table 1:** Distribution of subjects among quintile groups of municipality by population density

Category	Quintile of municipalities sorted by population density, no. (%)^a^						*P* value	RR^b^ of 1	95% CI
	1 low	2	3	4	5 high	Total			
PGY-1									
All graduates	1805 (20.7)	1842 (21.1)	1627 (18.7)	1855 (21.3)	1582 (18.2)	8711 (100.0)		1.000	NA–NA
Scholarship alone	54 (48.2)	35 (31.3)	10 (8.9)	9 (8.0)	4 (3.6)	112 (100.0)	< 0.001	2.327	1.912–2.831
Quota alone	34 (31.5)	21 (19.4)	24 (22.2)	18 (16.7)	11 (10.2)	108 (100.0)	0.082	1.519	1.147–2.013
Quota with scholarship	125 (51.0)	69 (28.2)	22 (9.0)	18 (7.3)	11 (4.5)	245 (100.0)	< 0.001	2.462	2.163–2.802
Jichi	52 (40.9)	42 (33.1)	17 (13.4)	9 (7.1)	7 (5.5)	127 (100.0)	< 0.001	1.976	1.597–2.445
PGY-2									
All graduates	1611 (19.6)	1721 (21.0)	1570 (19.1)	1704 (20.8)	1603 (19.5)	8209 (100.0)		1.000	NA–NA
Scholarship alone	60 (46.2)	28 (21.5)	14 (10.8)	13 (10.0)	15 (11.5)	130 (100.0)	< 0.001	2.352	1.943–2.846
Quota alone	46 (35.9)	25 (19.5)	32 (25.0)	10 (7.8)	15 (11.7)	128 (100.0)	< 0.001	1.831	1.447–2.317
Quota with scholarship	81 (43.8)	52 (28.1)	21 (11.4)	18 (9.7)	13 (7.0)	185 (100.0)	< 0.001	2.231	1.884–2.642
Jichi	40 (39.6)	33 (32.7)	14 (13.9)	8 (7.9)	6 (5.9)	101 (100.0)	< 0.001	2.018	1.580–2.578
PGY-3									
All graduates	1467 (17.9)	1510 (18.4)	1591 (19.4)	1722 (21.0)	1905 (23.2)	8195 (100.0)		1.000	NA–NA
Scholarship alone	54 (47.4)	33 (28.9)	13 (11.4)	9 (7.9)	5 (4.4)	114 (100.0)	< 0.001	2.646	2.169–3.229
Quota alone	27 (28.4)	21 (22.1)	20 (21.1)	12 (12.6)	15 (15.8)	95 (100.0)	0.070	1.588	1.150–2.192
Quota with scholarship	90 (49.7)	43 (23.8)	26 (14.4)	13 (7.2)	9 (5.0)	181 (100.0)	< 0.001	2.778	2.382–3.239
Jichi	89 (78.1)	9 (7.9)	8 (7.0)	1 (0.9)	7 (6.1)	114 (100.0)	< 0.001	4.361	3.916–4.857
PGY-4									
All graduates	1646 (21.1)	1547 (19.8)	1446 (18.5)	1646 (21.1)	1521 (19.5)	7806 (100.0)		1.000	NA–NA
Scholarship alone	59 (44.7)	49 (37.1)	15 (11.4)	6 (4.5)	3 (2.3)	132 (100.0)	< 0.001	2.120	1.745–2.575
Quota alone	39 (37.9)	20 (19.4)	19 (18.4)	16 (15.5)	9 (8.7)	103 (100.0)	0.001	1.796	1.397–2.308
Quota with scholarship	85 (62.5)	28 (20.6)	12 (8.8)	6 (4.4)	5 (3.7)	136 (100.0)	< 0.001	2.964	2.584–3.399
Jichi	98 (88.3)	7 (6.3)	4 (3.6)	2 (1.8)	0 (0.0)	111 (100.0)	< 0.001	4.187	3.864–4.537
PGY-5									
All graduates	1542 (20.9)	1467 (19.9)	1379 (18.7)	1565 (21.2)	1419 (19.2)	7372 (100.0)		1.000	NA–NA
Scholarship alone	57 (53.3)	24 (22.4)	9 (8.4)	8 (7.5)	9 (8.4)	107 (100.0)	< 0.001	2.547	2.121–3.058
Quota alone	26 (53.1)	7 (14.3)	6 (12.2)	9 (18.4)	1 (2.0)	49 (100.0)	< 0.001	2.537	1.942–3.313
Quota with scholarship	46 (65.7)	13 (18.6)	6 (8.6)	1 (1.4)	4 (5.7)	70 (100.0)	< 0.001	3.142	2.637–3.742
Jichi	89 (84.0)	7 (6.6)	4 (3.8)	3 (2.8)	3 (2.8)	106 (100.0)	< 0.001	4.014	3.653–4.411
Total									
All graduates	8071 (20.0)	8087 (20.1)	7613 (18.9)	8492 (21.1)	8030 (19.9)	40,293 (100.0)		1.000	NA–NA
Scholarship alone	284 (47.7)	169 (28.4)	61 (10.3)	45 (7.6)	36 (6.1)	595 (100.0)	< 0.001	2.383	2.186–2.598
Quota alone	172 (35.6)	94 (19.5)	101 (20.9)	65 (13.5)	51 (10.6)	483 (100.0)	< 0.001	1.778	1.574–2.007
Quota with scholarship	427 (52.3)	205 (25.1)	87 (10.6)	56 (6.9)	42 (5.1)	817 (100.0)	< 0.001	2.609	2.437–2.794
Jichi	368 (65.8)	98 (17.5)	47 (8.4)	23 (4.1)	23 (4.1)	559 (100.0)	< 0.001	3.287	3.086–3.500

*PGY* postgraduate year; *RR* relative risk; *CI* confidence interval

^a^Quintile 1 ≤ 522.7; quintile 2 ≤ 1301.0; quintile 3 ≤ 4665.3; quintile; 4 ≤ 10,492.6; quintile 5 > 10,492.6 persons per square kilometer

^b^Relative risk of Quintile 1 against Quintiles 2–5 with "all graduates" being the reference group

*P* value was calculated with Chi-square test with Bonferroni adjustment

The high likelihood of working in rural areas of Jichi, quota with scholarship, and scholarship alone were also observed in the non-metropolitan rate (Table 2). The tendency of higher rural likelihood in the longer PGY groups was also observed.

**Table 2:** Distribution of subjects between metropolitan and nonmetropolitan municipalities

Category	Metro^a^	Non metro^b^	Total	% of nonmetro	*P* value	RR^c^ of nonmetro	95% CI
PGY-1							
All graduates	5077	3634	8711	41.7	Reference	1.000	NA–NA
Scholarship alone	36	76	112	67.9	< 0.001	1.627	1.429–1.852
Quota alone	68	40	108	37.0	> 0.99	0.888	0.693–1.137
Quota with scholarship	108	137	245	55.9	< 0.001	1.340	1.196–1.502
Jichi	69	58	127	45.7	> 0.99	1.095	0.904–1.326
PGY-2							
All graduates	4938	3271	8209	39.8	reference	1.000	NA–NA
Scholarship alone	63	67	130	51.5	0.036	1.293	1.093–1.531
Quota alone	75	53	128	41.4	> 0.99	1.039	0.844–1.279
Quota with scholarship	94	91	185	49.2	0.051	1.234	1.064–1.433
Jichi	55	46	101	45.5	> 0.99	1.143	0.922–1.417
PGY-3							
All graduates	5233	2962	8195	36.1	Reference	1.000	NA–NA
Scholarship alone	53	61	114	53.5	< 0.001	1.480	1.245–1.761
Quota alone	56	39	95	41.1	> 0.99	1.136	0.891–1.448
Quota with scholarship	75	106	181	58.6	< 0.001	1.620	1.429–1.838
Jichi	19	95	114	83.3%	< 0.001	2.306	2.114–2.515
PGY-4							
All graduates	4559	3247	7806	41.6	Reference	1.000	NA–NA
Scholarship alone	48	84	132	63.6%	< 0.001	1.530	1.341–1.745
Quota alone	57	46	103	44.7%	> 0.99	1.074	0.865–1.333
Quota with scholarship	44	92	136	67.6%	< 0.001	1.626	1.444–1.832
Jichi	4	107	111	96.4%	< 0.001	2.317	2.216–2.423
PGY-5							
All graduates	4171	3201	7372	43.4%	Reference	1.000	NA–NA
Scholarship alone	41	66	107	61.7%	< 0.001	1.421	1.221–1.653
Quota alone	27	22	49	44.9%	> 0.99	1.034	0.757–1.412
Quota with scholarship	28	42	70	60.0%	0.031	1.382	1.139–1.676
Jichi	12	94	106	88.7%	< 0.001	2.042	1.899–2.197
Total							
All graduates	23,978	16,315	40,293	40.5%	Reference	1.000	NA–NA
Scholarship alone	241	354	595	59.5%	< 0.001	1.469	1.374–1.572
Quota alone	283	200	483	41.4%	> 0.99	1.023	0.919–1.138
Quota with scholarship	349	468	817	57.3%	< 0.001	1.415	1.332–1.503
Jichi	159	400	559	71.6%	< 0.001	1.767	1.675–1.865

*PGY* postgraduate year; *RR* relative risk; *CI* confidence interval; *Jichi* Jichi Medical University

^a^Metropolis: wards of the ordinance-designated cities, special wards of Tokyo, and center cities

^b^Nonmetropolis: other cities and towns/villages

^c^Relative risk of nonmetropolis against metropolis with "all graduates" being the reference group

*P* value was calculated with Chi-square test with Bonferroni adjustment

**Fig. 5:**
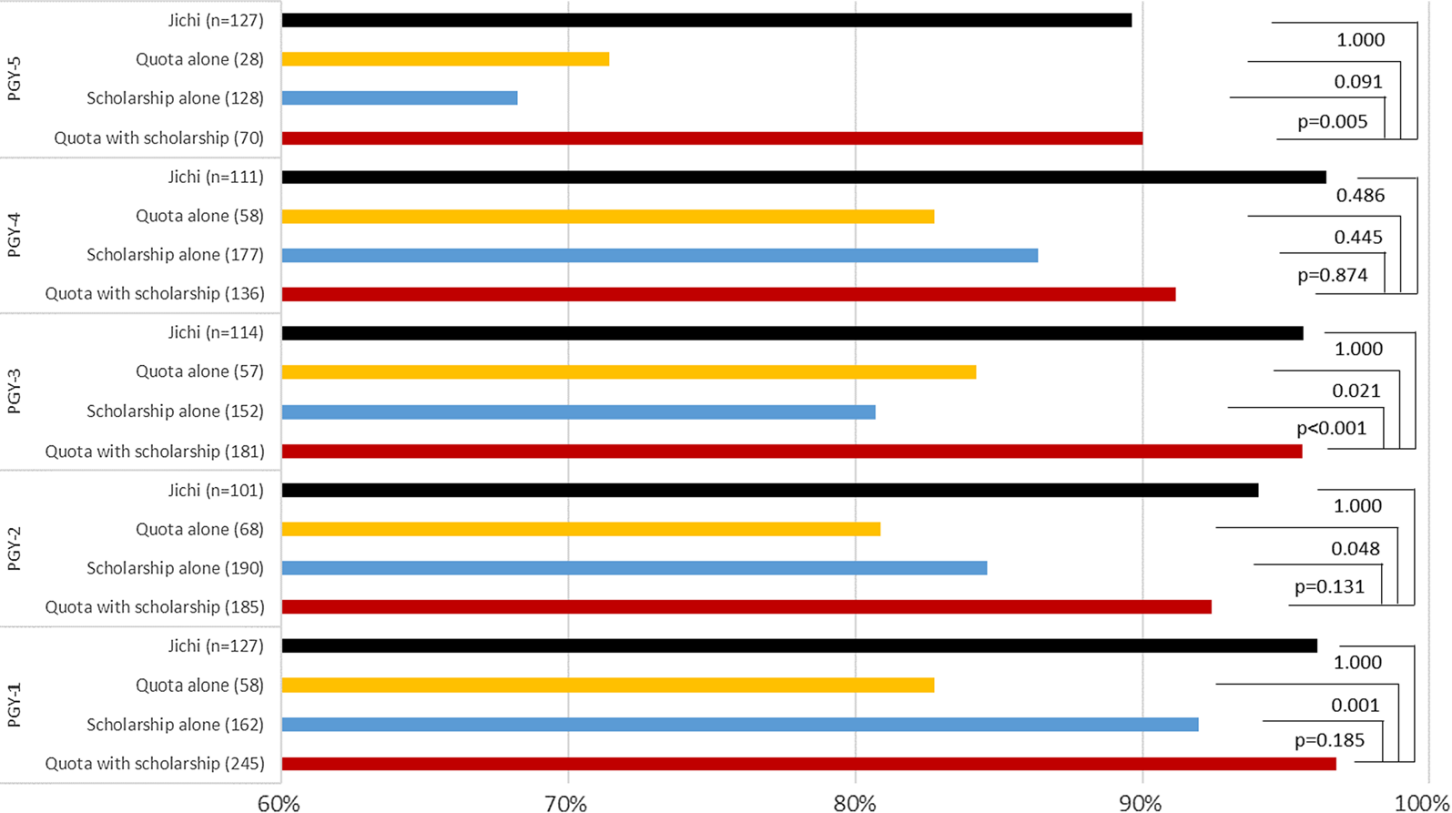
Proportion of subjects working in the designated prefecture

In terms of the in-prefecture rate (Fig. 5), Jichi and quota with scholarship were highest among the four groups and the rates of these two groups were statistically close, while the rate of quota alone was significantly lower than quota with scholarship in PGYs 1 to 3. No boost from the longer PGY was observed.

**Fig. 6:**
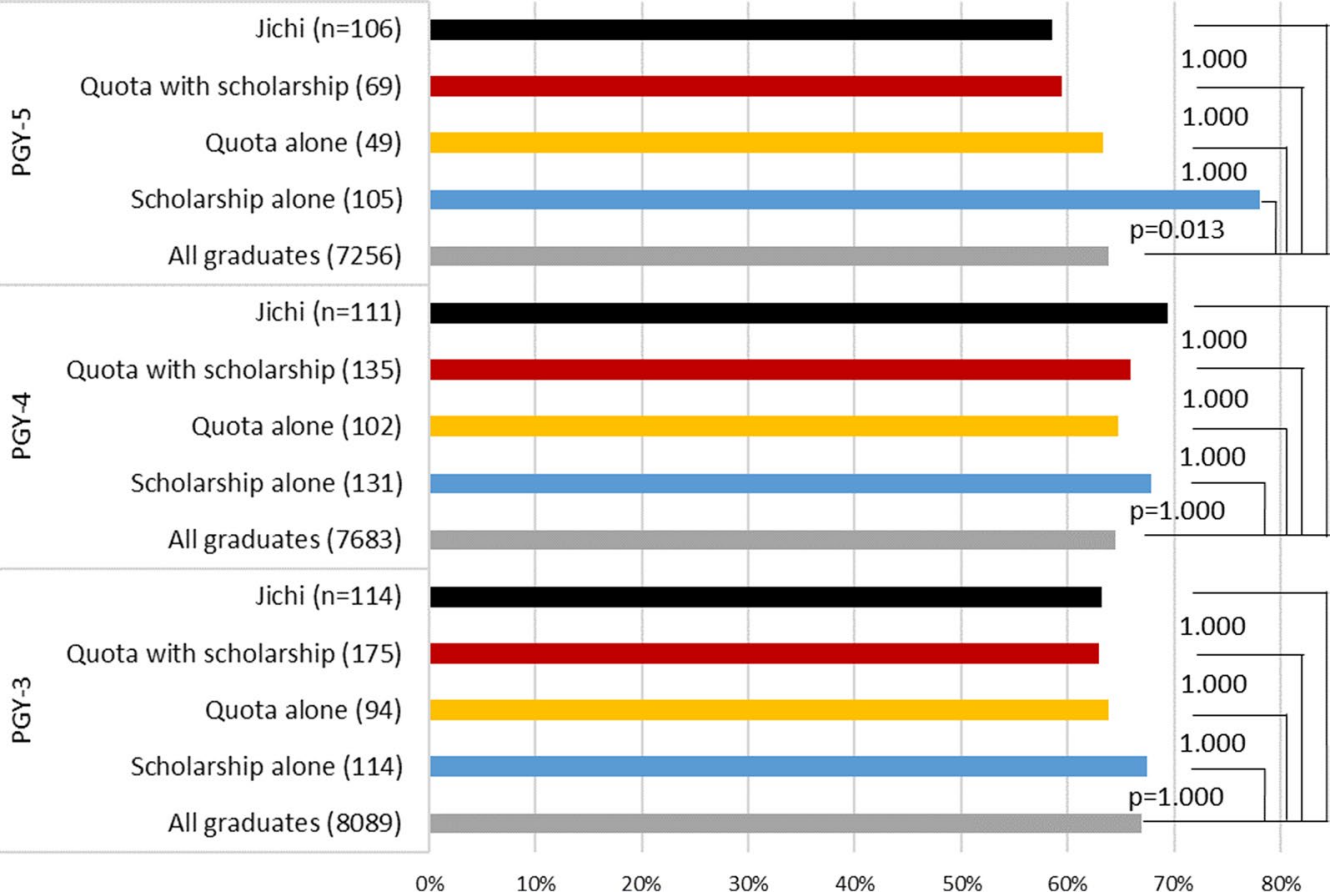
Proportion of subjects specialising in internal medicine or general practice

There was no significant difference between each program and all graduates in the proportion of internal medicine or general practice in PGYs 3 to 5 (Fig. 6).

## Discussion

Jichi, quota with scholarship, scholarship alone, and quota alone were effective in securing physician workforce in rural areas. The order of effectiveness was Jichi > quota with scholarship > scholarship alone > quota alone, and it became more obvious with the passage of time. This order is also held in terms of the results of physician license examination and compliance for obligatory rural service.

**Fig. 7:**
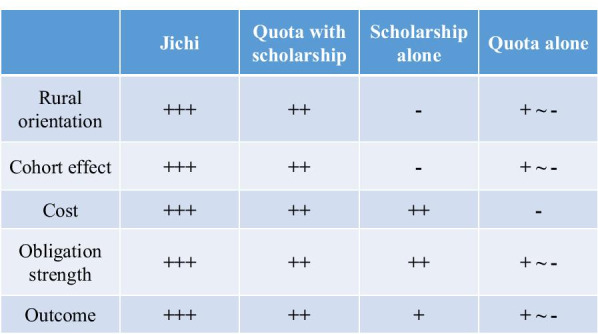
Summary of differences in outcome and features among programs. + means the program has the element;− means the program does not. The number of plus signs indicates the strength of the element

Figure 7 offers several reasons for this order. First, the degree to which the curriculum emphasizes rural health differs among programs. Jichi's curriculum is based on its special mission. Quota with scholarship is managed as a part of a medical school, so rural education is added as an extracurricular activity for quota students. Students of quota alone and scholarship alone usually do not have such a regular rural education. Second, the cohort effect created by classmates and similar-minded colleagues is different. In Jichi, all the students have obligatory rural service after graduation, so they can more easily maintain their motivation for the service, while students in quota with scholarship are a minority in the medical school class and so it is harder for them to remain motivated. Moreover, most of the students in quota alone or scholarship alone cannot benefit from this cohort effect, because they have no opportunity to meet regularly with their colleagues. Third, educational costs for each prefecture are different. The financial burden is heaviest in Jichi, for which prefectures must offer the whole budget to run a full-size medical school. For quota with scholarship and scholarship alone, each prefecture bears the cost only of scholarship and extracurricular rural education. Jichi graduates who breach the obligation must pay 23,000,000 yen (equivalent to US$219,000) though this refund still does not cover the full cost of their 6-year education [32, 33]. In contrast, graduates of quota with scholarship and scholarship alone must pay back 12,900,000 yen (US$123,000) on average, which is almost equivalent to the actual cost borne by the prefecture [18, 34]. Fourth, the strength of the obligation is different. Jichi graduates must spend about 5 years in rural areas of their home prefectures and usually are not permitted to have a deferral, compared to about 4 years with possible deferral for a few years for graduates of quota with scholarship. Graduates of quota alone do not have contractual, and, therefore, no legal, obligation to serve in any place. These differences might explain the inter-program gap in effectiveness shown in this study.

The chosen specialty is not different among programs. This is probably because these programs' main purpose is not to redress specialty distribution but to improve geographic distribution. Some prefectures restrict selectable specialties for Jichi and scholarship graduates or give an incentive to some specialties by exchanging them for rural service exemption. However, these initiatives are not large enough to change the specialty distribution of all graduates. As to the in-prefecture rate, Jichi and quota with scholarship were not different and both retained their graduates well in the designated prefectures. Reasons for a small number of graduates who worked in non-designated prefectures are, in addition to cases with a breach of obligation, cases with permitted leave due to marriage or specialty training.

The passing rate of those in quota with scholarship for the physician license examination was substantially higher than that of typical medical students, which are consistent with the results of a previous study [13]. This study also revealed that the passing rate of Jichi was even higher than that of quota with scholarship. This may be because the admission process in Jichi and many of the quota programs is a combined evaluation of the high school grade, extracurricular activity, recommendation letter, personal statement, interview and academic test score of the applicant, while in the usual admission process of medical schools the decision is based predominantly on the test score. The result suggests the personal aptitude for studying medicine assessed from multiple perspectives is better than that assessed solely based on the score of entrance examination, which casts doubt on the validity of the conventional admission process of Japanese medical schools.

Japanese health policy is rapidly shifting from the physician increase to the rectification of their maldistribution. The national government predicted a surplus of physicians, and is aiming to cut the enrolment capacity of medical schools [35], while declaring its intention to close urban–rural gap of physician supply by revising the Medical Care Act in 2019 [36]. This means that Jichi and quota with scholarship will be more important than before. In reality, the revised Medical Care Act requests each prefecture to provide a "career plan" so that the programs' graduates can balance specialty training and rural service, while authorizing the governor to participate in the decision making for the enrolment capacity of quota in a medical school. From a prefectural perspective, Jichi is a high cost/high return, quota with scholarship is a middle cost/middle return, and quota alone is a low cost/low return program. Each prefecture and medical school needs to understand the advantages and disadvantages of each program and use it rationally.

Many initiatives to recruit and retain physicians in rural areas are conducted around the world. In the United States, the federal government and state governments have return-of-service scholarship or loan forgiveness programs, such as National Health Service Corps (NHSC) [37–39]. There are many similar financial incentives in the world, and the estimated retention rate of the pooled subjects of these in contractual services was reportedly 71% in 2009 [27]. There are also many undergraduate programs for attracting medical students to rural practice in, for example, the United States, Canada, the Philippines, Thailand, Australia, New Zealand, Norway, and Scotland [25, 40–45]. These foreign programs usually focus on the preferential entry of students with a rural background and/or undergraduate primary care education in rural areas, and thereby attaining success in recruiting and retaining their graduates in rural areas. However, Jichi and quota with scholarship in Japan are different from these other initiatives in that they combine four elements: medical school admission, undergraduate rural-oriented education, scholarship, and obligatory rural service. Such a comprehensive education–scholarship initiative is also planned in South Korea. The South Korean government is planning to implement a special admission quota in medical schools to increase the number of medical students by 4000 (18%) over the next 10 years and send three quarters of them to rural provinces in exchange for tuition waivers and scholarships [20–22]. The results of this study will be a reference for the countries planning to introduce a new comprehensive education-scholarship program or to modify existing programs into more comprehensive ones. However, attention should be paid to the difference in the health system and culture when applying the results to societies outside Japan [46].

The nationwide and prospective nature of the data is an advantage of this study. Follow-up information is precise, because it derives from the legally enforced census data in case of quota and scholarship, and from the school institutional research data in Jichi. This study also covers almost 100% of potential subjects for analyses of passing rate for Physician License Examination and retention rate for the obligatory workforce.

There are some limitations to this study. Even though we accounted for 100% of Jichi graduates, we had 40% of graduates of quota with scholarship for geographic analyses. However, the timing of entry of the quota subjects to this cohort was 2 years before their actual placement to rural areas. It is, therefore, difficult to suppose that the response rate of potential subjects varies, depending on the rurality of their future workplace. In fact, a close look at the data shows that the response rate of each prefecture in 2014–2018 was not significantly correlated with its population density (Spearman correlation coefficient 0.000, *P* = 0.997.) Thus we consider the selection bias of study subjects is minimal.

## Conclusions

The Japanese medical education policies to produce rural physicians were effective. However, the degree of a program’s effectiveness varied greatly, depending on its curriculum, cost, and strength of obligation. The Japanese national and prefectural governments should plan their future rural workforce policies taking these variations into account. The results would be a reference for policy makers and medical educators in other countries as well which have adopted or are in the process of adopting the comprehensive education-scholarship program.

## Supplementary Information

**Additional file 1.** Supplementary text for the methodology of cross-sectional survey and cohort study.

## Data Availability

The data sets generated and analysed during the current study are not publicly available because they include personal data of participants but are available from the corresponding author on reasonable request. The data of Physician Census 2018 are available from Ministry of Health, Labour and Welfare but restrictions apply to the availability of these data, which were used under permission for the current study, and so are not publicly available.

## Abbreviations

Jichi: Jichi Medical University
Scholarship alone: Non-quota with scholarship
Quota alone: Quota without scholarship
EBPM: Evidence-based policy making
JCCME: The Japanese Council for Community-based Medical Education
Physician Census: Survey of Physicians, Dentists and Pharmacists 2018, Ministry of Health, Labour and Welfare
PGY: Postgraduate year
RR: Relative risk
CI: Confidence interval
NHSC: National Health Service Corps
