# Supplementary material for: Education policies to increase rural physicians in Japan: a nationwide cohort study
Source: Hum Resour Health. 2021 Aug 24;19:102. doi: 10.1186/s12960-021-00644-6 (PMC8386080; doi:10.1186/s12960-021-00644-6)
Supplement: Supplementary file 1 — Additional file 1. Supplementary text for the methodology of cross-sectional survey and cohort study. [file 12960_2021_644_MOESM1_ESM.docx]

**Supplementary Text**

**Annual cross-sectional survey of prefectures and medical schools**

Each June, the cohort office sends a questionnaire to each prefectural government and medical school to obtain information on the number of new graduates who had received scholarship (prefecture), who passed the National License Examination for Physicians (both), and who have bought out the scholarship (prefecture).

**Process of the cohort study**

As part of the survey, the study office asked each prefecture and medical school to send a questionnaire to all prospective study participants. The prefecture then administered the questionnaire, which asks the physician identification number of all quota and non-quota graduates with scholarship from the prefecture; the medical school sent the questionnaire to all of its quota graduates without scholarship. Each graduate then returned the completed questionnaire to the cohort office, which was registered as the cohort study's baseline data. All participants were newly licensed physicians. The Ministry of Health, Labour and Welfare; the Ministry of Education Culture, Sports, Science and Technology and the Association of Japan Medical Colleges supported this study by requesting the participation of prefectures and/or medical schools.

To add follow-up data to the cohort dataset, we connected individual baseline data, through physician identification number, to the Physician Census 2018. Japanese law requires all licensed physicians to register in the Census every two years. The Census gathers information on practice location, type of medical facility, work contents, specialty, and board certification status. In 2020, the Ministry of Health forwarded the data to the study office with special permission for this study (permission no 0601-1).

Among the 327,211 physicians registered in the Census which included both non-program physicians and cohort subjects, 40,292 licensed in 2014–2018 were extracted as “all graduates” and used as the control group.
